# Memorandum on Tetanus

**Published:** 1918-01

**Authors:** 


***Memorandum on Tetanus.*** By the War Office Committee on the Study of Tetanus. Abs. from the *Brit. Med. Jour.*, Nov. 11, 1916.

The Committee notes that after about 10 days the immunity from serum is to a great extent lost. A second preventive injection should, they believe, be made in all cases at the end of 7 days. If the wounds are severe, especially those from shell and bomb, as many as 4 injections should be given at intervals of 7 days. They think that danger from anaphylactic shock is negligible, if doses of 500 U. S. A. units contained in 3 cc. of horse serum are given subcutaneously, whatever the interval after a previous injection.

*Preventive Treatment. Dose.* All preventive injections should consist of 500 U.S.A. units (or 1/3 of the usual phial containing 1,500 units of antitoxin). It is not necessary to sterilize the syringe each time if a freshly sterilized needle is used.

*Operation.* A preventive dose of serum should be administered before any operation, unless the operation is performed within seven days of a previous injection. As tetanus is known to result from operations in the region of a wound several weeks after that wound has completely healed, a preventive injection should be given before all such operations. 500 units are sufficient in most cases; but, if administered subcutaneously, the injection, to insure complete absorption, should be made 2 days in advance; 12 hours are sufficient for the absorption of an intramuscular injection.

*Antiseptics.* The group of oxidizing antiseptics, such as hydrogen peroxide, potassium permanganate, chlorine water, and solution of iodine, are effective in checking the anaerobic growth of the tetanus bacillus. They have the power of rendering toxin non-toxic.

*Diagnosis.* The classical symptoms of tetanus, risus sardonicus and lockjaw, indicate a stage of the disease when treatment has lost much of its value. In men who have been treated prophylactically, trismus and the general symptoms practically never occur. Manifestations of tetanus are then confined to local spastic rigidity of the wounded limb, persisting sometimes for weeks. Early diagnosis is of utmost importance, since any delay in applying necessary treatment greatly diminishes the chances of success.

The toxin of tetanus does not advance through the blood but through the nerves in the region of the wound. It is quickly communicated to the spinal column and thence to the motor nerve cells governing the muscles in the neighborhood of the wound. It is therefore of the utmost importance that the slightest indication of rigidity or twitchings, or local increased reflex response to gentle tapping or pressure, be reported to the surgeon in charge. Nurses and attendants should be required to watch for such indications when the dressings are renewed and to report them at once.

Other early symptoms help in the diagnosis, such as, an anxious look, pain in the back of the neck, sore throat, general restlessness, unreasonable outbursts of temper, insomnia, violent headache, excessive yawning, complaints of spasm or stiffness in an injured limb, stiff neck or difficulty in swallowing without recognizable cause, stitch in the side, profuse local or general sweats, and difficulty in micturition.

*Treatment.* A delay of an hour in applying treatment may make all the difference between failure and success. Days or even weeks before a condition of trismus sets in, local indications, especially hardness and rigidity of the muscles around the wound, are usually to be observed; and the slightest symptom of the sort should be considered sufficient reason for specific treatment.

*Specific Treatment.* Since the intravenous injection of serum requires two days for complete absorption intrathecal injections are recommended as the first form of treatment.

The bed should be tilted up at the foot and the pillow removed for an hour or two after the injections.

*Dosage.* Very large doses are necessary for curative action. It is not wise, however, to inject more serum than will replace the amount of spinal fluid drawn off. This is usually not more than 20 cc. The injection should be made *very slowly.* The ordinary strength of the serum is 150 units to 1 cc. Thus 3,000 units may be administered. If a higher potency serum is available — say, 800 units to 1 cc. — it should by all means be used. Thus 16,000 units may be administered at a single injection. At the same time, 5,000 to 10,000 units should be injected intramuscularly, and 2,000 to 5,000 may be given subcutaneously.

The intrathecal injections should be continued daily from 3 to 5 days and then discontinued. Intramuscular or subcutaneous injections may be repeated daily or oftener, according to the severity of the symptoms. When the signs of abatement in the disease appear, the doses may be reduced in size and frequency, and the injections made only subcutaneously.

The following table of treatment has given good results for early, but well marked, tetanus :

| DAY | Subcutaneous | Intramuscular | Intrathecal |
|---|---|---|---|
| First day. . . . . . . . . . . . . . | — | 8,000 | 16,000 |
| Second day. . . . . . . . . . . . . | — | 8,000 | 16,000 |
| Third day. . . . . . . . . . . . . . | — | 4,000 | 8,000 |
| Fourth day. . . . . . . . . . . . . | — | 4,000 | 8,000 |
| Fifth day. . . . . . . . . . . . . . | 2,000 | — | — |
| Seventh day . . . . . . . . . . . . | 2,000 | — | — |
| Ninth day. . . . . . . . . . . . . . | 2,000 | — | — |

*Symptomatic Treatment.* — Such treatment consists in the exhibition of sedative drugs. Morphine in 1/4 gr. doses, administered every 4 hours, is the most suitable; potassium bromide, chloral, chloretone, paraldehyde are also given by the mouth or rectum.

*Carbolic acid* appears to have no curative effect whatever, nor any effect upon the course of the disease.

*Magnesium sulphate* has no effect upon the disease itself. Its effect in checking a spasm is only temporary and it is produced at considerable risk. There is probably no real advantage in its use.

*Surgical treatment.* — Excision of the wound and amputation, practiced as a means of checking the advance of tetanus, are now generally considered of little or no avail, if, indeed, they are not positively dangerous. It is therefore preferable to abstain from all operation, except when necessary for other reasons, until the usual treatment for tetanus has been carried out.

Irrigation of the wound with oxidizing agents, especially hydrogen peroxyde, is desirable, if it can be accomplished without opening up the wound.

